# Editorial

**Published:** 1952-07

**Authors:** 


### Editorial

### PULMONARY EMBOLISM

Once a month the medical staff of the United Bristol Hospitals meet to consider the recent deaths. One of the results of this practice is that the members quickly become aware of any striking changes in the incidence of fatal illnesses. Mr. M. G. Wilson's article in this issue is based on a survey of the hospital records that he undertook at the request of the medical staff who had become alarmed by the apparent increase in deaths from massive pulmonary embolism. The figures he now presents confirm their fears. Massive pulmonary embolism has become a sufficiently frequent hazard to justify a full investigation of the problem and the Board of Governors has appointed Mr. E. W. Parry, F.R.C.S., as a whole-time research assistant for this purpose.

The problem is not a new one and it is not confined to Bristol; in fact in some hospitals in the United States it is customary to tie the iliac veins, or even the inferior vena cava, as soon as femoral venous thrombosis is diagnosed, so as to prevent a pulmonary embolism.

A curious feature of the incidence of massive pulmonary embolism is that it appears to be commoner in some hospitals than in others. An excellent study of the condition in Southmead Hospital, Bristol, by Dr. D. S. Short in the *British Medical Journal* on April 12th, 1952, has shown a lower incidence of massive embolism in that hospital, although minor embolism and infarction were common. An annotation in the same journal says " It is now fully realized that fatal pulmonary embolism is a rare condition ": that unfortunately is not true everywhere—but why ? Many possible explanations of the recent increase come to mind: intravenous therapy, new anaesthetics and antibiotics; all these must be assessed. But the problem is really divisible into two parts—why venous thrombosis occurs and why the thrombus is liberated into circulation. Patients frequently have venous thrombosis in their lower limbs but mercifully few of them suffer embolism. Is it that venous thrombosis is now more frequent or is it that some agency causes the loosening of thrombi from their site of formation ? It will require a careful clinical study to evaluate the many procedures, such as leg exercises, designed to prevent thrombosis; and it is equally important to discover what factor favours liberation of the thrombus as a dangerous embolus. It may be just a question of how much propagation clot is formed in the large vein above the initial thrombotic site, for such clots are lightly attached, but it is perhaps more likely that some fibrinolytic agency is responsible for dissolving their attachment to the vessel walls. R. G. Macfarlane at Oxford has investigated the titre of naturally occurring fibrinolysin in the blood of patients before, during and after operation and has found an increase quite often before the operation begins ! He has tentatively correlated this with the observation that embolism was more frequent during the air raids. Can it be that alarm is responsible ? There is evidence that adrenalin increases the fibrinolyisn titre and here is a possible explanation of the association.

Several bacteria are known to produce active fibrinolysins. It is possible that infection of the blood stream by relatively non-pathogenic fibrinolysin-producing bacteria may be responsible for loosening thrombi.

It will be appreciated that Mr. Parry has a big field to investigate. The problem is real and urgent and the co-operation of all the medical and nursing staff will be needed for its solution.

### MEDICAL JOURNAL OF THE SOUTH-WEST

As part of the policy to enlarge this journal to represent medical activities in the whole South-West Region, the *Bristol Medico-Chirurgical Society* has decided to alter the title to *Medical Journal of the South-West.* At the same time the Law governing the composition of the editorial committee has been amended to permit the appointment of members from other societies and centres. We hope these steps will enable the Journal to become more closely associated with those in the Region outside the Bristol area.

The first number of the next volume in January, 1953, will appear under the new title.

